# Rapid FTIR Spectral Fingerprinting of Kidney Allograft Perfusion Fluids Distinguishes DCD from DBD Donors: A Pilot Machine Learning Study

**DOI:** 10.3390/metabo15110702

**Published:** 2025-10-29

**Authors:** Luis Ramalhete, Rúben Araújo, Miguel Bigotte Vieira, Emanuel Vigia, Ana Pena, Sofia Carrelha, Anibal Ferreira, Cecília R. C. Calado

**Affiliations:** 1 Blood and Transplantation Center of Lisbon, Instituto Português do Sangue e da Transplanta-ção, Alameda das Linhas de Torres, n◦ 117, 1769-001 Lisbon, Portugal; 2 Nova Medical School, Faculdade de Ciências Médicas, NMS, FCM, Universidade Nova de Lisboa, 1169-056 Lisbon, Portugal; 3 iNOVA4Health—Advancing Precision Medicine, Núcleo de Investigação em Doenças Renais, Nova Medical School, Faculdade de Ciências Médicas, NMS, FCM, Universidade Nova de Lisboa, 1169-056 Lisbon, Portugal; 4 ISEL—Instituto Superior de Engenharia de Lisboa, Rua Conselheiro Emídio Navarro 1, 1959-007 Lisbon, Portugal; 5 Instituto Politécnico de Lisboa, Estrada de Benfica Road 529, 1549-020 Lisbon, Portugal; 6 Nephrology, Hospital Curry Cabral, Unidade Local de Saúde de São José, R. da Beneficência 8, 1050-099 Lisbon, Portugal; 7 Hepatobiliopancreatic and Transplantation Center, Curry Cabral Hospital, Unidade Local de Saúde de São José, R. da Beneficência 8, 1050-099 Lisbon, Portugal; 8 Centro Clínico Académico de Lisboa, 1169-024 Lisbon, Portugal; 9 Institute for Bioengineering and Biosciences (iBB), The Associate Laboratory Institute for Health and Bioeconomy-i4HB, Av. Rovisco Pais, 1049-001 Lisbon, Portugal; 10 Instituto Superior Técnico (IST), Av. Rovisco Pais, 1049-001 Lisbon, Portugal; 11 Universidade de Lisboa (UL), Cidade Universitária, Alameda da Universidade, 1649-004 Lisbon, Portugal

**Keywords:** FTIR spectroscopy, kidney transplantation, perfusion fluid, DCD vs. DBD, machine learning

## Abstract

Background/Objectives: Rapid, objective phenotyping of donor kidneys is needed to support peri-implant decisions. Label-free Fourier-transform infrared (FTIR) spectroscopy of static cold-storage Celsior^®^ perfusion fluid can discriminate kidneys recovered from donation after circulatory death (DCD) versus donation after brain death (DBD). Methods: Preservation solution from isolated kidney allografts (*n* = 10; 5 DCD/5 DBD) matched on demographics was analyzed in the Amide I and fingerprint regions. Several spectral preprocessing steps were applied, and feature extraction was based on the Fast Correlation-Based Filter. Support vector machines and Naïve Bayes were evaluated. Unsupervised structure was assessed based on cosine distance, multidimensional scaling, and hierarchical clustering. Two-dimensional correlation spectroscopy (2D-COS) was used to examine band co-variation. Results: Donor cohorts were well balanced, except for higher terminal serum creatinine in DCD. Quality metrics were comparable, indicating no systematic technical bias. In Amide I, derivatives improved classification, but performance remained modest (e.g., second derivative with feature selection yielded an area under the curve (AUC) of 0.88 and an accuracy of 0.90 for support vector machines; Naïve Bayes reached an AUC of 0.92 with an accuracy of 0.70). The fingerprint window was most informative. Naïve Bayes with second derivative plus feature selection identified bands at ~1202, ~1203, ~1342, and ~1413 cm^−1^ and achieved an AUC of 1.00 and an accuracy of 1.00. Unsupervised analyses showed coherent grouping in the fingerprint region, and 2D correlation maps indicated coordinated multi-band changes. Conclusions: Performance in this 10-sample pilot should be interpreted cautiously, as perfect leave-one-out cross-validation (LOOCV) estimates are vulnerable to overfitting. The findings are preliminary and hypothesis-generating, and they require confirmation in larger, multicenter cohorts with a pre-registered analysis pipeline and external validation.

## 1. Introduction

Kidney transplantation continues to face a persistent supply–demand gap, prompting broader utilization of kidneys recovered from donors after circulatory death (DCD), i.e., from patients whose hearts irreversibly stopped and the consequent blood circulation has led to the cessation of oxygenation of the organism, including the organ to be collected. Across contemporary syntheses and registry analyses, DCD kidneys exhibit a consistently higher incidence of delayed graft function (DGF) than kidneys from donors after brain death (DBD), whereas medium- to long-term graft survival can converge under appropriate selection and preservation strategies [1,2]. This divergence, greater early functional vulnerability with eventual outcome convergence, creates a practical imperative: rapid, objective phenotyping before implantation to support triage, allocation, and peri-operative management [1,2,3].

Although both donor pathways culminate in ischemia–reperfusion injury (IRI), DCD and DBD follow distinct injury chronologies that plausibly shape early outcomes. DCD procurement necessarily entails a period of warm ischemia (variously defined as functional or total warm-ischemia time), with hypoxia, acidosis, endothelial activation, and microvascular stasis that heighten DGF risk [1,4,5,6]. By contrast, DBD is characterized by systemic neuro-hormonal and inflammatory perturbation, catecholamine surges, cytokine release, and activation of complement and coagulation pathways, with hemodynamic instability that can precondition the graft microenvironment, even in the absence of warm ischemia [1,7].

Treating DCD and DBD as interchangeable early-risk states risks obscuring meaningful biological heterogeneity. The phenotyping of the organ should be sensitive to donor-type specific chemistry rather than relying solely on a categorical label [1,2,3,8].

The current work aimed to discover biomarkers discriminating DCD from DBD based on the analysis of the static cold-storage (SCS) preservation solution effluent collected intraoperatively from kidneys flushed and stored in Celsior^®^ (Genzyme, Cambridge, MA, USA). This analysis was not conducted in hypothermic or normothermic machine perfusion perfusate conditions; instead, the preservation technique was widely used in routine kidney SCS practice. Furthermore, SCS remains prevalent globally; an assay that reads out directly from SCS effluent, therefore, has broad deployability across centers and procurement settings. Moreover, the effluent plausibly concentrates cell-free biochemical signals liberated during the vascular flush and cold-storage interval, signals that may encode donor-type differences in injury biology and hence early functional risk. While distinct from perfusion circuits, the same conceptual framework, leveraging a chemistry-rich fluid compartment for rapid risk readouts, extends naturally to hypothermic and normothermic machine perfusion, where biomarker signatures in perfusate and physiologic pump parameters repeatedly associate with DGF and early performance [3,9,10,11,12]. Imaging add-ons during ex vivo perfusion can contribute orthogonal information but are not universally accessible, reinforcing the value of fast, scalable biochemical phenotyping during preservation [11].

There is now consolidated evidence that preservation fluids carry actionable information. Untargeted metabolomics of kidney perfusate during hypothermic machine perfusion has identified patterns associated with DGF and allograft failure [9], and narrative/systematic reviews catalog candidate biomarkers, amino acids, energy, and redox intermediates that add predictive value when combined with pump parameters [10,11]. Complementing these targeted chemistries, label-free spectral fingerprints offer a route to rapid, low-burden readouts that can be standardized across workflows. Within this context, Fourier-transform infrared (FTIR) spectroscopy provides a minute-scale, consumable-free approach to biochemical phenotyping. In the mid-infrared, FTIR captures global molecular “fingerprints” from small-volume biofluids, encoding contributions from proteins (Amide I/II), lipids, carbohydrates, and phosphates, the biochemical classes expected to shift with warm ischemia, inflammatory activation, and IRI [13,14,15,16,17].

Our research group has already shown clinical promise of FTIR spectroscopy in renal transplantation, pointing out that serum spectra can predict cellular rejection [18] and even discriminate between cellular from antibody-mediated rejection [19]. The present work aims to evaluate whether FTIR spectroscopic fingerprints of SCS preservation solution effluent differ between kidneys from DCD and DBD using a rapid, economical, and high-throughput workflow. To our knowledge, this is the first application of label-free FTIR to static cold-storage Celsior^®^ effluent for discriminating DCD from DBD. Methodologically, we (i) aggregate technical replicates at the donor level to avoid leakage, (ii) use a standardized pipeline, and (iii) complement the supervised results with 2D-COS and conservative unsupervised distances. Operationally, the assay is minute-scale and consumable-free, facilitating adoption in routine SCS workflows. To achieve that goal, the following were evaluated: diverse spectral preprocessing techniques, i.e., Savitzky–Golay derivatives, baseline correction, and vector normalization; dimensionality reduction and filter-based feature selection (e.g., Fast Correlation-Based Filter); and supervised classification models.

## 2. Materials and Methods

### 2.1. Study Design and Participants

This exploratory, retrospective, cross-sectional pilot study included perfusion fluids from deceased-donor kidney allografts. Only allografts perfused with Celsior^®^ (Genzyme, Cambridge, MA, USA) were analyzed with isolated kidney allografts to minimize procedural heterogeneity. Donor demographic and clinical variables (age, sex, body mass index, comorbidities, and laboratory parameters) were used to assemble two comparable cohorts by donor type (DBD vs. DCD). Matching aimed to minimize between-group imbalances; variables with excessive missingness or poor comparability were excluded (e.g., smoking status and cause of death). The study was approved by the ULS São José Ethics Committee (approval No. 1215/2022).

#### Sample Size Justification

This was a pilot, proof-of-concept study aimed at feasibility and signal detection rather than formal hypothesis testing. No a priori sample size calculation was performed. The sample size (*n* = 10; 5 DCD and 5 DBD) was determined by consecutive availability within the study window and balanced matching on donor/organ characteristics to limit confounding. To mitigate overfitting in a small-*n* setting, all classifiers were evaluated with LOOCV. Findings are hypothesis-generating and will be validated in a larger, prospectively powered cohort.

### 2.2. FTIR Spectra Acquisition

Aliquots of 25 μL of sample were pipetted onto a 96-well Silicon plate and dehydrated for approximately 3.5 h in a vacuum desiccator (Vacuubrand, ME 2, Wertheim, Germany). Spectral data were collected using an FTIR spectrometer (Vertex 70, Bruker, Mannheim, Germany) equipped with a High-Throughput Screening eXTension (HTS-XT) accessory (Bruker, Ettlingen, Germany). Each spectrum was acquired in transmission mode between 400 and 4000 cm^−1^, with 32 co-added scans at a resolution of 2 cm^−1^. The first well of each plate was left empty, and its corresponding spectra were used as the background, according to the HTS-XT manufacturer’s instructions. All spectra were acquired on the same instrument with identical parameters. Sample positions alternated with DCD/DBD to minimize spatial and edge effects, and donor identifiers were masked during preprocessing and modeling. Plates were processed under identical environmental conditions and instrument configuration to limit inter-plate variability.

### 2.3. Spectral Quality Control

Spectral quality was assessed using four complementary metrics computed on donor-level mean spectra. Technical replicates were first aggregated per donor by averaging across scans after aligning wavenumber axes; all QC calculations were then performed on these donor-level spectra. (i) The Amide I signal-to-noise ratio (SNR) was defined as the maximum Amide I peak height (1600–1700 cm^−1^) after rubber band baseline correction divided by noise estimated in an off-band window (1800–1900 cm^−1^). All downstream unsupervised and supervised analyses likewise used the donor-level spectra to avoid information leakage. If that window contained insufficient points, a predefined fallback of 2000–2200 cm^−1^ was used; if needed, the global standard deviation (SD) of the baseline-corrected spectrum provided a last-resort estimate to avoid undefined values. (ii) Spike artifacts were quantified in the fingerprint region (900–1800 cm^−1^) using a first-difference derivative and a median absolute deviation (MAD) threshold (flagging points with |z| > 6) to capture narrow, acquisition-related spikes. (iii) Cosine similarity to the cohort median was calculated between each vector-normalized fingerprint spectrum and the cohort median (also vector-normalized) as a measure of spectral shape coherence and outlier screening. (iv) Baseline area fraction was computed in the fingerprint region as the area under the rubber band baseline divided by the raw spectral area (reported as %), serving as a baseline drift index.

QC metrics were reviewed a priori to define minimal acceptability (non-zero SNR with plausible noise, limited spike burden, high cosine similarity, and low baseline fraction). All included spectra met these criteria; thus, no donor was excluded on QC grounds. These procedures were implemented in Python (Version 3.13.1, NumPy, SciPy, pandas, matplotlib) with a convex hull (rubber band) baseline, fixed spectral windows (fingerprint 900–1800 cm^−1^; Amide I 1600–1700 cm^−1^; off-band 1800–1900/2000–2200 cm^−1^), and consistent vector normalization for shape-based comparisons.

### 2.4. Spectral Preprocessing, Feature Selection, and Multivariate Analysis

Demographic and clinical variables for the study populations were evaluated to assess potential confounding factors [20]. Continuous variables are summarized as the median (interquartile range [IQR]), given the small sample size; where approximately symmetric, we also reported the mean (standard deviation [SD]). Categorical variables are presented as counts (percentages). Appropriate tests, including the chi-square test and Mann–Whitney U test, were performed using GraphPad Prism version 8.0.2 for Microsoft Windows (GraphPad Software, San Diego, CA, USA).

Spectral preprocessing began with atmospheric interference correction (H_2_O/CO_2_) in OPUS^®^ v6.5 (Bruker, Bremen, Germany). All subsequent steps were performed in Orange Data Mining v3.39.0 (Bioinformatics Lab, University of Ljubljana, Slovenia) and included rubber band baseline correction (BC), Savitzky–Golay first- and second-derivative transformations (second-order polynomial, 15-point window), and vector normalization (VN). Spectral deconvolution was applied only in the Amide I region (1600–1700 cm^−1^) to resolve overlapping protein secondary structure bands, whereas the fingerprint region (900–1800 cm^−1^) was analyzed without deconvolution.

Unsupervised structure was investigated by multidimensional scaling (MDS) using cosine distances and hierarchical clustering analysis (HCA) with Ward’s linkage. In addition, 2D-COS was performed to examine synchronous and asynchronous band correlations, highlighting co-varying vibrational features within both spectral regions.

Supervised classification relied on support vector machines (SVMs) and Naïve Bayes. Multiple preprocessing pipelines were tested: BC, VN, first derivative, first derivative with VN, second derivative, and second derivative with feature selection. Feature selection was carried out with the Fast Correlation-Based Filter (FCBF), which retains non-redundant, discriminative wavenumbers. Model performance was evaluated using LOOCV.

**Figure A1:**
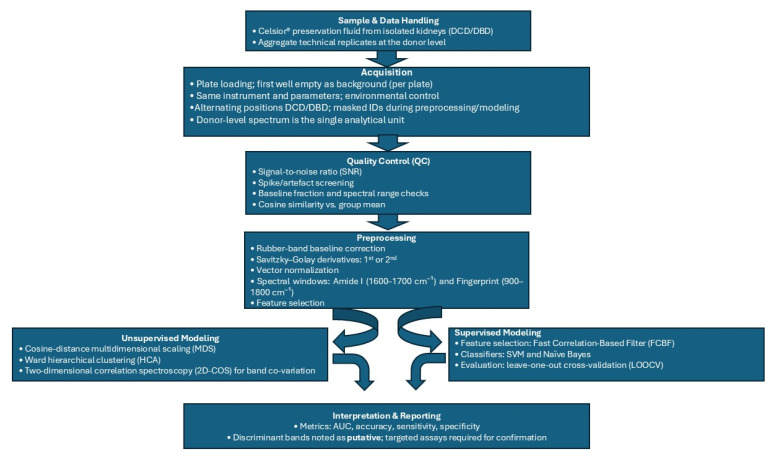
End-to-end FTIR workflow for kidney allograft preservation fluid analysis. Sample and acquisition (Celsior^®^ fluid; per-plate background; identical instrument settings; alternating DCD/DBD positions; masked IDs) → donor-level data handling (aggregate technical replicates) → quality control (SNR, spike screening, baseline fraction, cosine similarity) → preprocessing (rubber band baseline correction, Savitzky–Golay derivatives, vector normalization; Amide I 1600–1700 cm^−1^ and fingerprint 900–1800 cm^−1^ windows) → unsupervised analyses (cosine distance MDS, Ward HCA; 2D-COS for band co-variation) → supervised modeling (FCBF feature selection; SVM/Naïve Bayes; LOOCV; metrics: AUC, accuracy, sensitivity, specificity; 95% exact binomial CI for accuracy only) → interpretation/reporting (putative band assignments, pilot framing, plan for pre-registered external validation with stability/permutation tests and nested CV).

Because this pilot includes only 10 donors, we report 95% exact binomial confidence intervals for overall accuracy only; class-wise and AUC intervals were not computed because interval estimates are unstable at this sample size. For the same reason, we did not perform feature stability bootstraps or label permutation tests. A prospectively powered study will pre-register bootstrap-based stability analysis, permutation testing, and nested cross-validation to better calibrate optimism. The full preprocessing and analysis workflow is summarized in Appendix A, Figure A1.

## 3. Results

### 3.1. Baseline Characteristics of the Donor Cohorts

This exploratory study included perfusion fluids from deceased-donor kidney allografts. Only allografts perfused with Celsior^®^ (Genzyme, Cambridge, MA, USA) were analyzed with isolated kidney allografts to minimize procedural heterogeneity. Donor demographic and clinical variables, presented in Table 1 (age, sex, body mass index, comorbidities, and laboratory parameters), were used to assemble two comparable cohorts by donor type (DBD vs. DCD). Both cohorts were predominantly female (80% in DCD and 80% in DBD), which minimizes sex-related confounding between groups but limits generalizability; potential sex-specific effects are, therefore, considered in the Discussion. The study was approved by the ULS São José Ethics Committee (approval No. 1215/2022).

### 3.2. FTIR Spectra Quality

A quality control (QC) assessment was conducted to verify that all spectra were suitable for downstream preprocessing and classification. Four complementary metrics were evaluated: (i) Amide I SNR, computed as the maximum Amide I peak height after rubberband baseline correction divided by noise estimated in an off-band window (1800–1900 cm^−1^, with predefined fallbacks applied if that window was insufficient); (ii) spike artifact count in the fingerprint region (900–1800 cm^−1^), detected using a first-derivative median absolute deviation threshold; (iii) cosine similarity between each vector-normalized fingerprint spectrum and the cohort median spectrum as a measure of spectral shape coherence; and (iv) baseline area fraction in the fingerprint window, defined as the ratio of rubber band baseline area to raw spectral area and used as a drift index. Per-donor values are shown in Table 2, and group-level summaries (median [IQR], mean ± SD) with inferential statistics are reported in Table 3.

**Figure 1:**
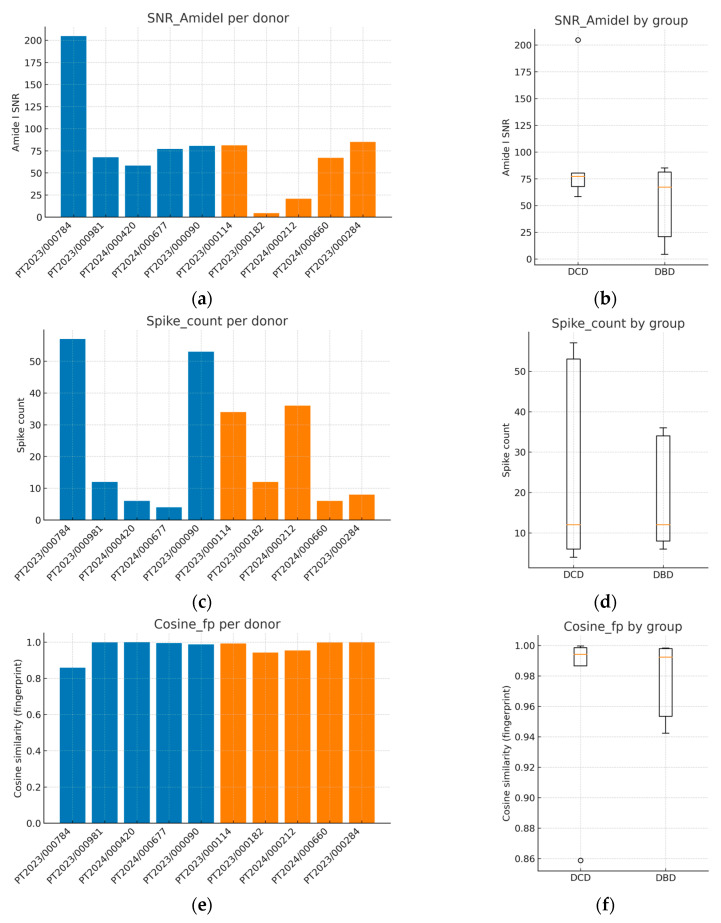
Quality control metrics of FTIR spectra from kidney allograft perfusion fluids. (**a**) The Amide I signal-to-noise ratio (SNR) per donor; (**b**) boxplot of SNR comparing DCD and DBD groups; (**c**) spike artifact counts per donor; (**d**) boxplot of spike counts by group; (**e**) cosine similarity in the fingerprint region per donor; (**f**) boxplot of cosine similarity by group. These QC analyses confirm adequate spectral quality across all donors, with consistently high cosine similarity (>0.95), low spike counts, and acceptable SNR, supporting the reliability of subsequent preprocessing and classification steps. Blue corresponds to donation after DCD and orange to donation after DBD. In box plots, the central line indicates the median, boxes represent the interquartile range (IQR), whiskers extend to 1.5 × IQR, and individual points denote donors.

Figure 1 summarizes the QC behavior both at the sample level (bar plots; panels a, c, e) and at the group level (box plots; panels b, d, f). Amide I SNR displayed inter-donor variability, including one low-SNR and several high-SNR cases, but all samples remained within analytically acceptable ranges for reliable interpretation of the Amide I structure (Figure 1a,b; Table 2). Spike artifacts were generally sparse, with modest heterogeneity across donors (Figure 1c,d). Cosine similarity values in the fingerprint region were consistently high for most spectra, indicating strong coherence of spectral shape across donors (Figure 1e,f). Baseline area fractions clustered near zero for all samples (≈0% of raw area), confirming negligible baseline drift; because values were effectively at the floor, a dedicated baseline fraction panel was not included.

Between-group comparisons using two-sided Mann–Whitney U tests with rank-biserial effect sizes did not identify statistically significant differences in any QC metric (Table 3). SNR did not differ between DCD and DBD donors (*p* ≈ 0.55), spike counts were indistinguishable (*p* ≈ 1.00), and cosine similarity was likewise comparable (*p* ≈ 0.69). These findings argue against systematic QC bias favoring either donor type and indicate that subsequent preprocessing, feature selection, and classification are unlikely to be confounded by differential spectral quality. Collectively, the QC profile, adequate SNR, low spike burden, high spectral coherence, and negligible baseline drift support the robustness of the dataset for the supervised analyses presented later.

### 3.3. Spectral Classification Performance

Supervised classification was evaluated in the Amide I (1600–1700 cm^−1^) and fingerprint (900–1800 cm^−1^) windows across multiple preprocessing pipelines (baseline correction, vector normalization, first/second Savitzky–Golay derivatives, and second derivative combined with FCBF) using support vector machines (SVMs) and Naïve Bayes under LOOCV. Summary metrics, such as AUC, accuracy, sensitivity, and specificity, are reported in Table 4. Accordingly, we report 95% CIs for overall accuracy (see Methods and Table 4).

In relation to the Amide I region, the SVM models trained on raw, baseline-corrected, or vector-normalized spectra performed close to chance (AUC 0.20–0.28; accuracy 0.50). Discrimination improved once band sharpening was introduced. The second derivative alone reached an AUC of 0.60, and adding FCBF further increased performance to an AUC of 0.88 and an accuracy of 0.90, with 0.70 for both sensitivity and specificity. Naïve Bayes models on this region showed the same trend, peaking with second derivative plus FCBF (driven by a band near ~1673 cm^−1^) at an AUC of 0.92 and an accuracy of 0.70 (0.70/0.70). These findings indicate that Amide I contains useful but limited donor-type signal unless derivatives and redundancy filtering are applied (Table 4).

In relation to the fingerprint (900–1800 cm^−1^) region, the discrimination models strengthened markedly. The SVM improved with derivatives, achieving an AUC of 0.84 and an accuracy, sensitivity, and specificity of 0.90 with the second derivative plus FCBF. Naïve Bayes was consistently strong. Vector normalization alone yielded an AUC of 0.76, where derivative pipelines reached an AUC between 0.86 and 0.88. The best overall Naïve Bayes model was based on second derivative spectra plus FCBF, which selected bands at ~1202, ~1203, ~1342, and ~1413 cm^−1^ and achieved an AUC of 1.00 (Table 4). This pattern suggests that composite biochemical content in the fingerprint region (including lipids, carbohydrates, phosphates, and proteins) carries the most informative signature for donor-type separation.

**Figure 2:**
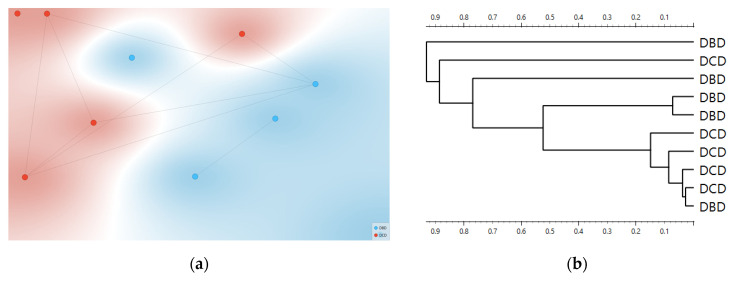
Distance-based exploration analysis of second derivative FTIR spectra. Cosine distance heatmap with hierarchical clustering (**a**) and multidimensional scaling (MDS) configuration (**b**). Donor groups (DCD in red vs. DBD in blue) show distinct clustering tendencies in the fingerprint region.

**Figure 3:**
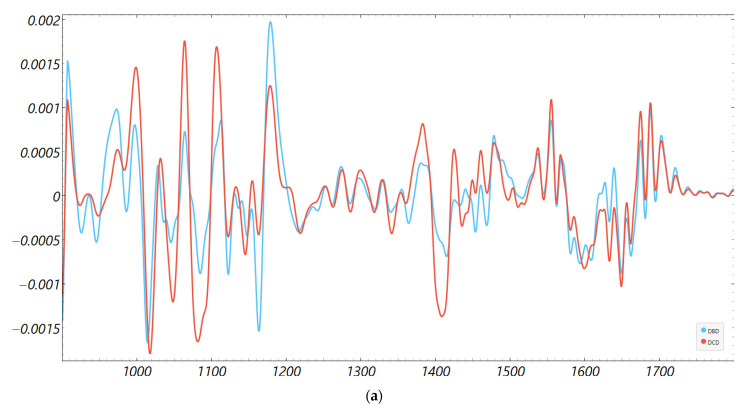

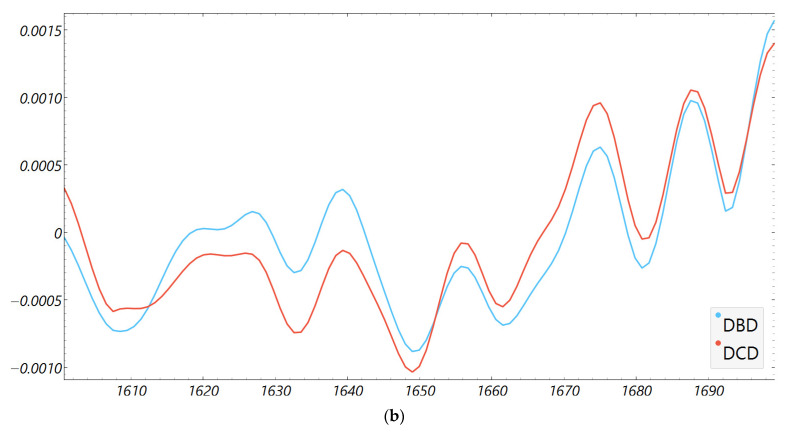
Second derivative average FTIR spectra of donor groups. Mean spectra for DCD and DBD perfusion fluids in the (**a**) fingerprint (900–1800 cm^−1^) and (**b**) Amide I (1600–1700 cm^−1^) regions.

**Figure 4:**
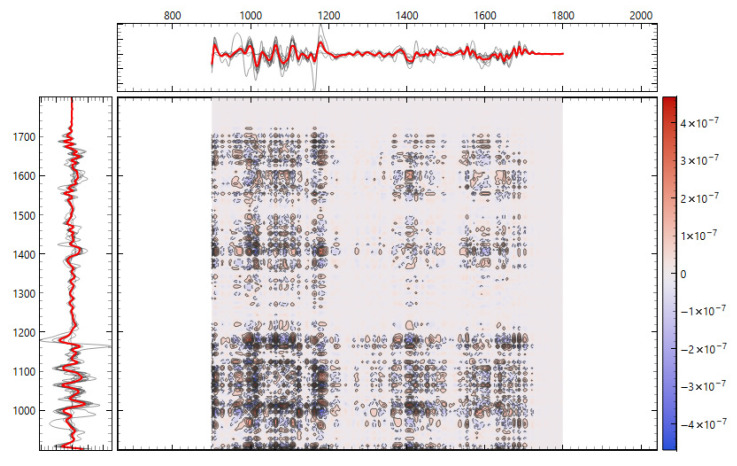

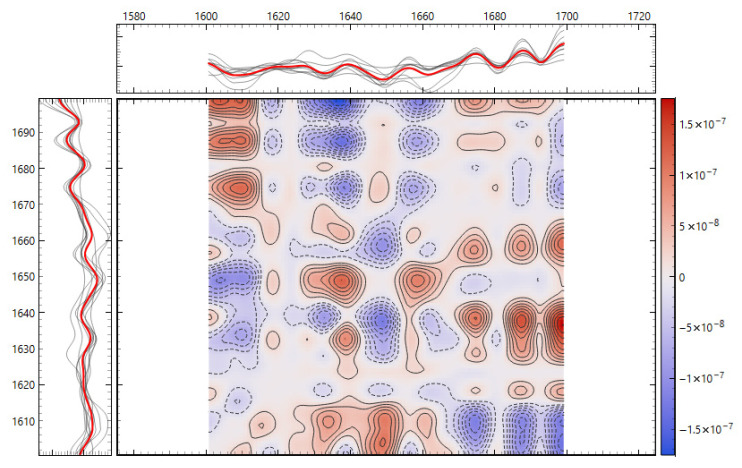
Two-dimensional correlation spectroscopy of second derivative FTIR spectra. (**top**) Synchronous 2D correlation map of the fingerprint region (900–1800 cm^−1^). (**bottom**) Synchronous 2D correlation map of the Amide I region (1600–1700 cm^−1^). Cross-peaks highlight co-varying vibrational bands, with intensity indicating the strength and direction of spectral correlations between donor groups.

Consistency with unsupervised structure and spectral organization was also observed. Distance-based exploratory analyses (Figure 2) support the supervised results. The cosine distance heatmap with hierarchical clustering and the corresponding MDS layout show clear group tendencies in the fingerprint region after derivative preprocessing, without obvious outliers. Group mean spectra (Figure 3) highlight macroscopic differences in the ~1200–1415 cm^−1^ range and around ~1673 cm^−1^, aligning with FCBF-selected features in the highest-performing pipelines. Synchronous 2D correlation maps (Figure 4) further corroborate coordinated behavior among these fingerprint bands and more moderate relationships within Amide I, indicating that discrimination arises from structured co-variation across multiple bands rather than a single isolated peak. These band assignments are putative and cannot be linked conclusively to specific biochemical pathways without targeted assays; orthogonal validation (e.g., liquid chromatography–mass spectrometry panels) will be required to confirm the molecular contributors.

An apparent discrepancy between AUC and accuracy in isolated pipelines (e.g., acceptable accuracy with a very low or inverted AUC) can occur in small cohorts when the score orientation is reversed relative to the positive class; this is a threshold/labeling artifact and does not contradict separability. Conversely, AUC/accuracy = 1.00 under LOOCV with *n* = 10 should be interpreted as proof of concept pending validation in larger, independent cohorts. Notably, the QC analysis (Section 3.2) showed comparable SNR, spike burden, and spectral coherence across groups, reducing the likelihood that technical artifacts drive the observed classification behavior.

## 4. Discussion

This proof-of-concept study indicates that label-free FTIR spectroscopy of Celsior^®^ (Genzyme, Cambridge, MA, USA) preservation fluid contains a donor-type signal that can separate DCD from DBD when spectra are rigorously preprocessed and feature selection is conducted with FCBF. Across windows, the fingerprint (900–1800 cm^−1^) consistently outperformed the Amide I (1600–1700 cm^−1^) region, with the best pipeline (second derivative plus FCBF, Naïve Bayes) reaching excellent LOOCV performance in this small, matched cohort (Table 4). These findings are biologically plausible, concordant with current transplant evidence on donor pathway biology and with more studies showing that preservation fluids carry clinically actionable chemistry for peri-implant risk assessment [9,21].

In terms of biological plausibility and alignment with prior knowledge, DCD and DBD follow distinct injury chronologies before procurement. DCD entails warm ischemia with hypoxia/acidosis and microvascular stasis, whereas DBD is characterized by a catecholamine/cytokine storm and systemic inflammatory activation. These upstream differences plausibly imprint distinct biochemical fingerprints in the fluid that bathes the organ during flush and cold storage. Contemporary registry and cohort syntheses confirm that DCD has higher DGF, while medium-term survival converges with DBD under modern selection and preservation practices, which is precisely the scenario where fast, pre-implant phenotyping could add value [2,22,23].

The fingerprint window integrates lipids, carbohydrates, phosphates, and protein side-chain modes, making it sensitive to composite changes expected from ischemia–reperfusion and inflammatory signaling; by contrast, Amide I predominantly reflects protein backbone structure and, alone, is less discriminative unless bands are sharpened and redundancy is reduced. The specific wavenumbers highlighted by FCBF in our best models (~1202, ~1203, ~1342, ~1413 cm^−1^ in the fingerprint and ~1673 cm^−1^ in Amide I) fall within commonly reported biochemical assignments for these classes, supporting physiological coherence without over-attribution to single molecules [14,15].

Positioning relative to perfusion-based assessments. Although our matrix is SCS effluent rather than hypothermic machine perfusion/normothermic machine perfusion (HMP/NMP) perfusate, the conceptual framework is shared. Chemistry in preservation fluids tracks graft condition and early function. Multicenter work in untargeted perfusate metabolomics linked specific molecules to death-censored graft failure years later, and systematic reviews catalog perfusate biomarkers that complement pump parameters for DGF prediction [9,21].

In parallel, randomized evidence shows that end-ischemic NMP after SCS reduces DGF in DCD, underscoring the potential impact of timely biochemical readouts on peri-implant choices. A tiered workflow is thus plausible, with QC-gated FTIR as an ultrafast, consumable-free screen, followed (if flagged) by targeted assays or short NMP for functional testing, and finally integrative models that combine spectral features, perfusate biomarkers, and pump metrics [12].

There are methodological considerations that explain the performance pattern. In small-cohort biofluid spectra, Savitzky–Golay derivatives (second order, fixed window) are standard to resolve overlapping bands and minimize the impact of baselines/slopes; VN mitigates pathlength/concentration effects; rubber band BC addresses broad background; and feature selection (e.g., FCBF) retains informative, non-redundant wavenumbers. The superiority observed for the second derivative pipeline and the additional gain from FCBF are consistent with best practice in clinical FTIR spectroscopy. The 2D-COS maps (synchronous) reinforce that discrimination results from coordinated co-variation among diverse bands, particularly in the fingerprint, rather than a single band, which is desirable for generalizability [15,24,25,26].

When interpreting performance metrics, appropriate safeguards should be applied. First, in a small population (*n* = 10), occasional discordance between the AUC and accuracy can arise from score orientation (e.g., if the model’s decision scores are inverted relative to the “positive” class). This flips the receiver operating characteristic (ROC) and the AUC without changing the confusion matrix. This highlights the relevance of reporting both discrimination and threshold metrics. Second, the AUC and accuracy of 1.00 with LOOCV points to learnability within this cohort, but this must be treated as a proof of concept pending external validation and/or larger-sample cross-validation (CV) (e.g., nested CV or label permutation tests). These cautions are standard in small-sample spectroscopy and are consistent with consensus protocols for bio-IR spectral analytics [27,28].

Observations concerning QC parity and the creatinine imbalance are as follows. Our QC (SNR, spike burden, cosine similarity, baseline fraction) showed no significant group differences between spectral data, arguing against a technical bias driving classification (Section 3.2). The higher terminal creatinine in DCD (with concordant estimated glomerular filtration rate trend) is clinically expected, and warm ischemia and hemodynamic instability are predisposed to donor acute kidney injury (AKI) prior to retrieval. Importantly, contemporary kidney transplant studies show that elevated terminal creatinine or donor AKI does not preclude acceptable outcomes when selection is judicious; several analyses report similar long-term graft survival despite higher DGF and more complex early courses. Thus, while creatinine may partly mediate spectral differences (e.g., via protein catabolism or tubular injury chemistry leaking into effluent), it does not invalidate the observed separation; rather, it highlights a plausible biological conduit through which donor pathway injury is encoded in the spectra. Future work should quantify this explicitly (e.g., regress selected wavenumbers on creatinine, stratify by AKI stage, or repeat matching within narrow creatinine bands) [2,29,30]. Both groups in this pilot were 80% female, reducing cross-group confounding but limiting external generalizability. While sex differences can influence transplant outcomes and injury biology, the balanced distribution across DCD and DBD in our cohort makes a sex-driven separation between donor types less likely here. Larger cohorts will explicitly stratify by sex and test interaction terms (e.g., sex × donor type) or include sex as an adjustment factor to quantify any sex-specific effects on spectral signatures and classification performance.

The fingerprint captured the most robust signal because it aggregates mixed biochemical classes susceptible to ischemia- and inflammation-driven remodeling: lipid peroxidation and membrane turnover (e.g., CH and phosphate bands), glyco-signatures from extracellular matrix turnover, and Amide III/CH deformations from proteins. The Amide I window, while informative for a secondary structure, is narrower and more sensitive to water/CO_2_ and pathlength nuances; in our data, it required derivatives and feature filtering to reach high performance. This regional asymmetry is well-described in clinical FTIR spectroscopic studies of biofluids and tissues and matches our observation that group means ± SD diverged visibly in ~1200–1415 cm^−1^ and to a lesser degree around ~1673 cm^−1^ (Figure 3 and Figure 4) [14,27,28,31].

Why does unsupervised structure matter here? We deliberately avoided t-Distributed Stochastic Neighbor Embedding (t-SNE), unstable in small *n*, and used cosine MDS and Ward HCA, which showed consistent group tendencies in the fingerprint after derivative preprocessing (Figure 2). This triangulation is important. When supervised models say “separable,” and unsupervised distances also reveal coherent clustering without outliers, while QC is comparable, the risk that a hidden technical confounder drives the effect is reduced. The 2D-COS maps add orthogonal evidence by highlighting band-to-band correlations aligned with the features selected by FCBF [25].

Regarding limitations and generalizability, it should be emphasized that this was an exploratory, single-center study with a small sample size (*n* = 10). Although LOOCV is reasonable at this scale, it still risks optimistic estimates; nested CV, bootstrap, and label-permutation testing would better calibrate small-sample uncertainty. The current acquisition used a standardized HTS transmission workflow with internal open-beam backgrounds; despite near-zero baseline fractions, subtle film-thickness heterogeneity cannot be excluded. Using solvent blanks or matrix-matched references and external standards will further tighten variance, though VN and second derivatives already mitigate much of it (and our QC did not favor one group). Finally, the current work is intentionally restricted to Celsior^®^ (Genzyme, Cambridge, MA, USA) SCS effluent and isolated kidneys to reduce procedural heterogeneity; transferability to HMP/NMP and to other solutions requires direct validation. The literature suggests that biomarker-rich perfusates under HMP/NMP are predictive and that brief end-ischemic NMP improves early DCD outcomes, so FTIR spectroscopy could complement rather than replace those assessments in a tiered clinical pathway [12,21].

Clinical applicability and next steps are as follows. FTIR offers a minute-scale, consumable-free screen that fits seamlessly within routine SCS workflows and can be run at the back table before implantation. A pragmatic pathway involves (i) rapid FTIR screening during SCS; (ii) for FTIR-flagged grafts, escalation to targeted assays (e.g., predefined perfusate panels) or a short end-ischemic bout NMP for functional readouts; and (iii) integration of spectral features with perfusion metrics into a simple decision rule to inform triage, allocation, and peri-implant management. This tiered approach prioritizes speed and scalability, reserving resource-intensive steps for higher-risk grafts and aligning with emerging evidence on perfusate analytics and ex situ perfusion trials. To move beyond a proof of concept, we will pre-register a fixed pipeline (windows, derivative parameters, VN, FCBF, classifier) and test it prospectively in multicenter cohorts using nested cross-validation with permutation, calibration transfer across instruments, and sensitivity analyses quantifying the contribution of creatinine/AKI to spectral separation. We will also benchmark against state-of-the-art perfusate panels and pump parameters to position FTIR relative to the current best practice. If validated, FTIR could function as a low-burden front end that narrows downstream testing to the subset of grafts most likely to benefit from targeted chemistry or short NMP, consistent with the direction of recent randomized and observational evidence in kidney perfusion science [8,9,12,21,32,33].

Finally, these results reaffirm established guidance for clinical bio-IR: standardize derivative order and spectral windows, avoid over-tuning, use VN and BC consistently, report both discrimination and threshold metrics, and prefer conservative unsupervised checks (MDS/HCA) over visually seductive but unstable embeddings at small *n*. As clinical applications of FTIR grow, following consensus protocols remains essential for reproducibility and safe translation [27].

In summary, FTIR profiling of Celsior^®^ preservation fluid distinguished DCD from DBD in a small, balanced pilot study. The results demonstrate feasibility and generate hypotheses, but they should not be over-interpreted given *n* = 10 and LOOCV-based estimates. A pre-registered pipeline, independent external validation, and stability/permutation analyses in larger cohorts are needed to assess generalizability.

## 5. Conclusions

This work is a pilot, hypothesis-generating study of label-free FTIR spectroscopy applied to Celsior^®^ (Genzyme, Cambridge, MA, USA) preservation effluent from DCD and DBD kidney donors. Using standardized preprocessing (rubber band baseline correction, Savitzky–Golay derivatives, vector normalization) and filter-based feature selection (FCBF), we observed donor-type separation driven mainly by the fingerprint window (900–1800 cm^−1^), with Amide I (1600–1700 cm^−1^) contributing complementary protein-dominated signal after derivative sharpening. In this small, matched cohort (*n* = 10), the best pipeline—second derivative with FCBF on Naïve Bayes models—achieved excellent performance under LOOCV, while distance-based unsupervised views and 2D correlation maps supported coordinated, multi-band differences rather than single-band effects.

Given the very limited sample size and the reliance on LOOCV, these results should be interpreted as a preliminary proof of concept rather than definitive evidence of clinical performance. Although QC metrics were balanced across groups, terminal creatinine was higher in DCD, as clinically expected, and could partly mediate spectral differences; this requires explicit sensitivity analyses in larger datasets. The main role of these findings is to show feasibility. FTIR spectroscopy can deliver a fast (minutes), consumable-free readout that integrates naturally with static cold-storage workflows and has the potential to inform donor phenotyping before implantation.

The next steps should move beyond this pilot; we will conduct multicenter external validation using a pre-registered, fixed pipeline (predefined spectral windows, derivative parameters, normalization, feature selection, and classifier choice). Evaluation will include rigorous cross-validation with label permutation, calibration transfer across instruments and sites, and matrix blanks/external standards to monitor drift and reproducibility. We will pair discriminant wavenumbers with targeted chemistry (e.g., liquid chromatography–mass spectrometry panels) to assign molecular contributors and assess biological plausibility. Reporting will include donor-level performance with confidence intervals, confusion counts, and decision curve analyses. If these preliminary signals are confirmed prospectively, FTIR profiling of preservation effluent can function as a rapid, scalable front end within routine SCS, delivering a minute-scale screen that prioritizes targeted assays or short NMP only for flagged, higher-risk grafts, thereby supporting triage, allocation, and peri-implant decision-making. We will also address governance and reproducibility (pre-registration, protocol sharing, and data/code availability) to facilitate adoption across centers.

## Figures and Tables

**Table 1:** The demographic and clinical characteristics of the two groups.

Feature	DBD (*n* = 5)	DCD (*n* = 5)	*p-Value*
Age	54.0 [54.0–64.0]	53.0 [50.0–54.0]	0.397615 ^1^
Sex (% female)	80%	80%	1 ^2^
Weight	70.0 [65.0–70.0]	74.0 [74.0–80.0]	0.137564 ^1^
Height	165.0 [160.0–165.0]	172.0 [165.0–175.0]	0.167938 ^1^
Body mass index	25.7 [25.4–25.7]	25.0 [24.2–29.4]	0.834035 ^1^
Hypertension (% yes)	60%	80%	1 ^3^
Diabetes	80%	80%	1 ^3^
Serum creatinine	0.6 [0.6–0.7]	1.1 [1.0–1.1]	0.045866 ^1^
Urea	21.0 [14.0–27.0]	35.0 [22.0–36.0]	0.150794 ^1^
Estimated glomerular filtration rate	93.0 [90.0–103.0]	77.0 [60.0–79.0]	0.059327 ^1^
Cardiorespiratory arrest (%yes)	40%	100%	0.166667 ^3^

DBD, donation after brain death; DCD, donation after circulatory death; ^1^ Mann–Whitney U test; ^2^ chi-square test; ^3^ Fisher’s exact test; two-sided α = 0.05.

**Table 2:** FTIR spectra quality control metrics for individual donor samples. Reported values include the Amide I signal-to-noise ratio (SNR), spike artifact counts, and cosine similarity to the fingerprint region median spectrum.

DonorID	Group	SNR_AmideI	Spike_Count	Cosine_fp
PT2023/000784	DCD	204.725	57	0.859
PT2023/000981	DCD	67.697	12	0.998
PT2024/000420	DCD	58.219	6	1
PT2024/000677	DCD	77.158	4	0.994
PT2023/000090	DCD	80.408	53	0.987
PT2023/000114	DBD	81.152	34	0.992
PT2023/000182	DBD	4.364	12	0.942
PT2024/000212	DBD	20.737	36	0.953
PT2024/000660	DBD	67.108	6	0.998
PT2023/000284	DBD	85.07	8	0.998

DBD, donation after brain death; DCD, donation after circulatory death.

**Table 3:** Comparison of FTIR quality control metrics between DCD and DBD donor groups. Data are presented as median [IQR] and mean ± SD. Group differences were assessed with two-sided Mann–Whitney U tests. Rank-biserial correlation (r) is reported as an effect size.

Variable	DCD_Median [IQR]	DBD_Median [IQR]	DCD_Mean ± SD	DBD_Mean ± SD	U	*p-Value*	Rank-Biserial_r
SNR_AmideI	77.16 [67.70, 80.41]	67.11 [20.74, 81.15]	97.64 ± 60.49	51.69 ± 36.80	16	0.5476	−0.28
Spike_count	12.00 [6.00, 53.00]	12.00 [8.00, 34.00]	26.40 ± 26.31	19.20 ± 14.60	13	1	−0.04
Cosine_fp	0.99 [0.99, 1.00]	0.99 [0.95, 1.00]	0.97 ± 0.06	0.98 ± 0.03	15	0.6905	−0.2

DBD, donation after brain death; DCD, donation after circulatory death.

**Table 4:** FTIR classification performance (LOOCV) for DCD vs. DBD across spectral regions, preprocessing pipelines, and classifiers.

Model	Region	Preprocessing	AUC	Accuracy (95% CI)	Sensitivity	Specificity
SVM	Amide I 1600–1700 cm^−1^	Rubber band BC	0.28	0.50 (0.19–0.81)	0.50	0.50
		VN	0.20	0.50 (0.19–0.81)	0.50	0.50
		1st derivative	0.32	0.30 (0.07–0.65)	0.30	0.30
		1st derivative + VN	0.24	0.70 (0.35–0.93)	0.41	0.70
		2nd derivative	0.60	0.80 (0.44–0.97)	0.60	0.80
		2nd derivative + FCBF (~1673 cm^−1^)	0.88	0.90 (0.55–1.00)	0.70	0.70
Naïve Bayes		Rubber band BC	0.70	0.50 (0.19–0.81)	0.50	0.50
		VN	0.56	0.70 (0.35–0.93)	0.70	0.70
		1st derivative	0.48	0.50 (0.19–0.81)	0.50	0.50
		1st derivative + VN	0.62	0.70 (0.35–0.93)	0.70	0.70
		2nd derivative	0.80	0.80 (0.44–0.97)	0.60	0.90
		2nd derivative + FCBF (~1673 cm^−1^)	0.92	0.70 (0.35–0.93)	0.70	0.70
SVM	Fingerprint 900–1800 cm^−1^	Rubber band BC	0.36	0.00 (0.00–0.31)	0.00	0.00
		VN	0.20	0.30 (0.07–0.65)	0.30	0.30
		1st derivative	0.00	0.70 (0.35–0.93)	0.70	0.70
		1st derivative + VN	0.08	0.40 (0.12–0.74)	0.40	0.40
		2nd derivative	0.64	0.70 (0.35–0.93)	0.70	0.70
		2nd derivative + FCBF (~1202, ~1203, ~1342, ~1413 cm^−1^)	0.84	0.90 (0.55–1.00)	0.90	0.90
Naïve Bayes		Rubber band BC	0.56	0.50 (0.19–0.81)	0.50	0.50
		VN	0.76	0.80 (0.44–0.97)	0.80	0.80
		1st derivative	0.74	0.70 (0.35–0.93)	0.70	0.70
		1st derivative + VN	0.86	0.80 (0.44–0.97)	0.80	0.80
		2nd derivative	0.88	0.80 (0.44–0.97)	0.80	0.80
		2nd derivative + FCBF (~1202, ~1203, ~1342, ~1413 cm^−1^)	1.00	1.00 (0.69–1.00)	1.00	1.00

BC, rubber band baseline correction; VN, vector normalization; FCBF, Fast Correlation-Based Filter. Accuracy 95% CI by exact binomial (Clopper–Pearson); *n* = 10 (LOOCV predictions). Class-wise and AUC CIs were not computed due to instability at this sample size.

## Data Availability

The data presented in this study is available upon request from the corresponding author.

## Abbreviations

The following abbreviations are used in this manuscript:

FTIR	Fourier-transform infrared spectroscopy
DCD	Donation after circulatory death
DBD	Donation after brain death
SCS	Static cold storage
2D-COS	Two-dimensional correlation spectroscopy
AUC	Area under the curve
IRI	Ischemia–reperfusion injury
VN	Vector normalization
BC	Rubber band baseline correction
FCBF	Fast correlation-based filter
LOOCV	Leave-one-out cross-validation
QC	Quality control
IQR	Interquartile range
SD	Standard deviation
ROC	Receiver operating characteristic
t-SNE	t-distributed stochastic neighbor embedding
